# Role of Trimetazidine in Ameliorating Endothelial Dysfunction: A Review

**DOI:** 10.3390/ph17040464

**Published:** 2024-04-05

**Authors:** Yusof Kamisah, Hamat H. Che Hassan

**Affiliations:** 1 Department of Pharmacology, Faculty of Medicine, Universiti Kebangsaan Malaysia, Kuala Lumpur 56000, Malaysia; kamisah_y@ppukm.ukm.edu.my; 2 Department of Medicine, Faculty of Medicine, Universiti Kebangsaan Malaysia, Kuala Lumpur 56000, Malaysia

**Keywords:** endothelial dysfunction, endothelium, atherosclerosis, apoptosis, angiogenesis

## Abstract

Endothelial dysfunction is a hallmark of cardiovascular diseases, contributing to impaired vasodilation, altered hemodynamics, and atherosclerosis progression. Trimetazidine, traditionally used for angina pectoris, exhibits diverse therapeutic effects on endothelial dysfunction. This review aims to elucidate the mechanisms underlying trimetazidine’s actions and its potential as a therapeutic agent for endothelial dysfunction and associated cardiovascular disorders. Trimetazidine enhances vasodilation and hemodynamic function by modulating endothelial nitric oxide synthase activity, nitric oxide production, and endothelin-1. It also ameliorates metabolic parameters, including reducing blood glucose, mitigating oxidative stress, and dampening inflammation. Additionally, trimetazidine exerts antiatherosclerotic effects by inhibiting plaque formation and promoting its stability. Moreover, it regulates apoptosis and angiogenesis, fostering endothelial cell survival and neovascularization. Understanding trimetazidine’s multifaceted mechanisms underscores its potential as a therapeutic agent for endothelial dysfunction and associated cardiovascular disorders, warranting further investigation for clinical translation.

## 1. Introduction

Cardiovascular diseases remain a leading cause of mortality and morbidity globally, representing a significant public health challenge [1]. Central to the pathophysiology of cardiovascular diseases is endothelial dysfunction, a condition characterized by impaired endothelial cell function and integrity [2]. Endothelium, lining the inner surface of blood vessels, plays a critical role in maintaining vascular homeostasis by regulating vascular tone, blood flow, and thrombosis [3]. Endothelial dysfunction disrupts these vital functions, leading to endothelial barrier dysfunction, proinflammatory state, oxidative stress, and impaired nitric oxide (NO) bioavailability [4,5,6]. Consequently, endothelial dysfunction contributes to various cardiovascular diseases, including hypertension, atherosclerosis, coronary artery disease, and peripheral vascular disease [2]. Understanding the mechanisms underlying endothelial dysfunction is essential for developing effective strategies to prevent and treat cardiovascular diseases.

**Figure 1:**
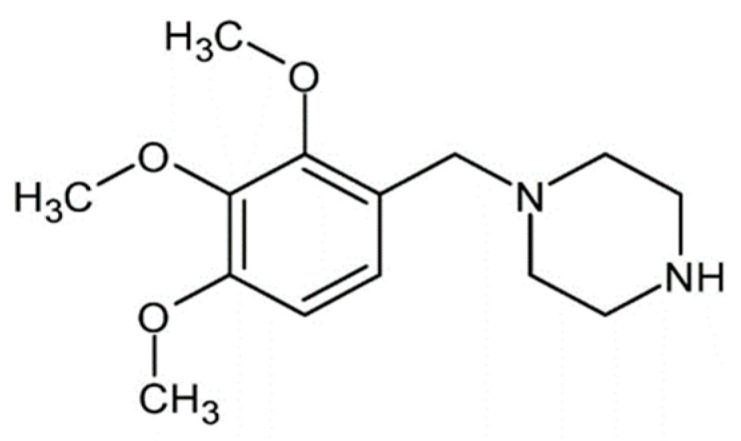
The molecular structure of trimetazidine.

Trimetazidine (Figure 1), traditionally used in the management of angina pectoris [7], is renowned for its efficacy as an anti-ischemic agent [8]. Recently, it has garnered attention for its potential therapeutic use beyond its primary indication. Emerging evidence indicates promising effects of trimetazidine on endothelial dysfunction, a pivotal pathological feature in various cardiovascular diseases. Its multifaceted mechanisms of action, including enhancing vasodilation [9], attenuating oxidative stress and inflammation [10], and modulating apoptosis and angiogenesis [11], along with its ability to reduce atherosclerosis [12] and blood glucose levels—a condition known to exacerbate endothelial dysfunction [10]—underscore its potential as a therapeutic agent. Given its pleiotropic effects on endothelial function, trimetazidine holds promise as a therapeutic agent for endothelial dysfunction and may offer additional benefits in the prevention and treatment of cardiovascular diseases.

However, the use of trimetazidine is not devoid of side effects. It commonly presents relatively mild gastrointestinal discomfort, including nausea and vomiting [13]. Previous reports have suggested a potential association between trimetazidine use and the incidence of Parkinson’s disease, based on case reports and small patient series [14,15]. Nevertheless, a clinical trial involving over 6000 patients who underwent percutaneous coronary intervention revealed that the incidence of Parkinson’s disease in the group taking trimetazidine was similar to that in the placebo group. Additionally, there was no observed association of trimetazidine intake with hepatic disorders, agranulocytosis, or thrombocytopenia [16].

This review aims to delve into the current understanding of trimetazidine’s impact on endothelial dysfunction and its potential therapeutic implications in cardiovascular disease management. By elucidating its mechanistic actions, this exploration seeks to pave the way for future research endeavors and therapeutic interventions.

## 2. Pharmacological Treatment of Endothelial Dysfunction

Various pharmacological agents are utilized to alleviate endothelial dysfunction, with research focusing on their potential to target this condition, aiming to improve underlying pathophysiology and decrease the risk of cardiovascular diseases. Notably, statins are prominent among these agents due to their primary function of reducing cholesterol levels. Moreover, statins exert pleiotropic effects on endothelial function by exhibiting anti-inflammatory and antioxidant properties, thereby diminishing the formation of reactive oxygen species (ROS) and offering protection against oxidized low-density lipoprotein (oxLDL) through Rac1 inhibition. They also enhance NO synthesis in the endothelium by upregulating endothelial nitric oxide synthase. However, it is essential to consider that high-intensity statins, such as rosuvastatin and atorvastatin, may increase the risk of diabetes [17,18]. 

Another class of drugs utilized for endothelial dysfunction is calcium channel blockers. The medications induce vasodilation by inhibiting the influx of calcium ions into vascular smooth muscle cells [19]. Furthermore, studies indicate that these blockers stimulate the production of endothelial NO [20]. Nonetheless, their primary side effects are linked to hypotension, encompassing systemic hypotension, palpitations, and pedal edema [19]. 

Angiotensin-converting enzyme (ACE) inhibitors and angiotensin receptor blockers (ARBs) are commonly employed to alleviate endothelial dysfunction by antagonizing the renin–angiotensin–aldosterone system, thereby reducing vasoconstriction and oxidative stress [21]. ACE inhibitors such as enalapril, ramipril, and quinapril impede the conversion of angiotensin I to angiotensin II, consequently mitigating the effects of angiotensin II, which enhance sodium and water retention via aldosterone stimulation, as well as reducing endothelin-1 (ET-1) secretion. Moreover, they inhibit the degradation of bradykinin, a vasodilator [22,23]. On the other hand, ARBs like losartan, irbesartan, and telmisartan elicit similar effects to ACE inhibitors by blocking the angiotensin receptor, without impacting angiotensin I conversion [23]. Common side effects associated with both drug classes include headache, fatigue, and back pain [24]. However, coughing is more frequently reported with ACE inhibitors [25].

Glucose-lowering medications, including sodium–glucose co-transporter 2 (SGLT2) inhibitors and dipeptidyl peptidase 4 (DPP4) inhibitors, have shown promise in enhancing endothelial function [26]. Specifically, SGLT2 inhibitors such as empagliflozin have been observed to mitigate endothelial dysfunction by preserving the integrity of the glycocalyx and restoring the mechanotransduction capacity of endothelial cells affected by glycocalyx damage [27]. Additionally, DPP4 inhibitors like vildagliptin alleviate endothelial dysfunction by upregulating endothelial NO synthase (eNOS) and exerting anti-inflammatory effects [28]. Glucagon-like peptide receptor agonists, another class of hypoglycemic agents, may also alleviate endothelial dysfunction by stimulating adenylate cyclase and promoting cyclic AMP synthesis, alongside their antioxidant and anti-inflammatory properties [18]. 

NO donors are also employed to induce vasodilation in cases of endothelial dysfunction. These agents encompass nitroglycerin, isosorbide nitrates, and sodium nitroprusside. By releasing NO into the vascular system, they prompt augmented vascular capacitance and vasodilation [29,30]. However, it has been reported that organic nitrates may paradoxically lead to endothelial dysfunction, attributed to the excessive production of highly reactive peroxynitrite, a byproduct of the reaction between NO and superoxide anion [30].

Despite the availability of major therapeutic options, there is still a need for novel interventions targeting endothelial dysfunction. Trimetazidine is not currently utilized clinically for this purpose. Nevertheless, emerging research suggests its potential as a therapy for this clinical problem.

## 3. Effects of Trimetazidine on Endothelium-Dependent Vasodilation and Hemodynamic Parameters

The endothelium plays a pivotal role in regulating vascular function, governing essential aspects such as vascular tone, blood flow, and permeability. In response to various physiological stimuli, it releases vasoactive compounds, notably NO and ET-1. NO, synthesized by eNOS, serves as a potent vasodilator, facilitating vessel relaxation [31]. Conversely, ET-1 exerts vasoconstrictor effects, opposing vasodilation and influencing vascular tone [32]. Hemodynamic parameters, including blood pressure and vascular resistance, are intricately linked to endothelial function, particularly vasodilation, with several cardiovascular risk factors associated with endothelium-dependent vasodilation [33]. Impaired vasodilatory capacity leads to endothelial dysfunction, contributing to abnormalities in hemodynamic parameters such as hypertension. Conversely, hemodynamic disturbances, such as elevated blood pressure, can exacerbate endothelial dysfunction, perpetuating a cycle of impaired vasodilation and vascular dysfunction.

Trimetazidine has been observed to enhance acetylcholine-stimulated endothelium-dependent dilation while not affecting nitroglycerin-induced vasorelaxation in patients with ischemic heart disease and left ventricular dysfunction, a phenomenon linked with increased oxygen consumption and improved heart function (Table 1) [34]. Additionally, it demonstrates an improvement in vasorelaxation during exercise and an augmentation of flow-mediated dilation [9,35], possibly attributed to its potential to enhance NO release. It increases NO synthesis by activating protein kinase B (Akt), leading to upregulation of its downstream substrate eNOS. Increased expression of eNOS would elevate NO synthesis, hence its level. In the presence of an Akt inhibitor, the protective effects of trimetazidine were abolished, confirming the involvement of Akt/eNOS [36]. Nevertheless, its impact on blood NO levels appears inconsistent across studies, hinting at alternative mechanisms of action (Table 2) [37,38]. It did not significantly affect NO levels in patients with unstable angina undergoing perioperative percutaneous coronary intervention [38] or in patients with lower extremity arteriosclerosis obliterans when combined with alprostadil, a prostaglandin known for its platelet aggregation suppression properties [37]. Hence, it could be hypothesized that the vasodilatory effects of the drug are mediated by mechanisms other than solely increasing NO bioavailability. Alternative pathways might involve other vasoactive substances such as angiotensin II, dopamine, and norepinephrine. Nonetheless, research on the effects of trimetazidine on these substances remains scarce.

Trimetazidine elevates endothelial cyclic guanosine monophosphate (cGMP) levels [43]. This increase is attributed to the stimulation of cGMP production by NO, which activates the enzyme guanylate cyclase. Acting as a second messenger, cGMP mediates the relaxation effects of NO on smooth muscle cells, facilitating vasodilation [44]. The heightened release of endothelial cGMP indirectly suggests increased NO availability. However, the consistent impact of trimetazidine on this vasoactive substance remains uncertain. Notably, when combined with berberine, a traditional Chinese medicine, trimetazidine demonstrated an increase in plasma NO levels, which correlated with elevated expression of the eNOS gene [9]. Berberine is recognized for its anti-inflammatory properties and its ability to enhance eNOS expression, thereby augmenting NO levels [45]. Consequently, the combination of trimetazidine and berberine may exert a synergistic effect.

NO is highly susceptible to oxidative stress, leading to decreased levels due to its inactivation [46]. Trimetazidine may not directly shield NO against oxidative stress. Its antioxidant and anti-inflammatory properties likely contribute to its promotion of endothelial relaxation in patients with cardiovascular conditions [9,33,34,35,37,41]. It is plausible that trimetazidine facilitates vasorelaxation through pathways beyond NO release. Moreover, it demonstrates beneficial effects on both plasma and endothelial ET-1 levels in diverse cardiovascular diseases, resulting in reduction in the peptide levels [42,43]. Its protective mechanisms may also involve endothelium-derived hyperpolarizing factors or prostacyclin. Further investigation is warranted to explore its impact on potassium channel opening and muscarinic receptors in smooth muscle cells.

To date, only one study has compared the effects of trimetazidine with another drug targeting endothelial dysfunction, namely ranolazine [41]. Ranolazine is a new antianginal medication that shares structural similarities with trimetazidine but possesses a different mechanism of action—it suppresses the late phase of the inward sodium current [19]. Both drugs demonstrated similar vasodilation effectiveness in terms of baseline diameter, hyperemia-induced diameter, baseline blood flow, percentage of flow-mediated dilation, and nitroglycerine-induced dilation after 12 weeks of treatment in patients with chronic ischemic heart disease [41]. However, further studies comparing the effectiveness of trimetazidine with other vasodilators should be conducted.

## 4. Effects of Trimetazidine on Glucose Metabolism

Hyperglycemia, clinically seen in diabetes mellitus, promotes endothelial dysfunction which occurs due to an imbalance in the production of vasoconstrictors and vasodilators, affecting both macrovasculature and microvasculature [47]. The rise in extracellular glucose level elevates its uptake across endothelial cells, leading to enhanced glucose metabolism via the polyol pathway. In the pathway, increased formation of sorbitol from glucose occurs due to elevated activities of aldose reductase [48]. Accumulation of sorbitol results in cellular changes leading to endothelial dysfunction [49]. 

Diabetic patients with cardiovascular disease who received 20 mg of trimetazidine three times daily demonstrated a reduction in blood glucose levels (Table 3) [33,42,43]. It is important to note that trimetazidine does not directly decrease blood glucose levels. Instead, its primary mechanism involves inhibition of 3-ketoacyl coenzyme A thiolase, an enzyme involved in mitochondrial fatty acid oxidation. This inhibition shifts cellular metabolism towards glucose metabolism [50]. Increased cellular glucose utilization consequently promotes a decrease in circulating glucose levels [51]. Trimetazidine increases forearm glucose uptake, total body glucose disposal (M value), and oxidation for energy production and reduces basal forearm non-glucose glycolysis, leading to decreased fasting blood glucose and insulin levels [42,43]. Additionally, the reduction in fasting insulin levels could be indicative of improved pancreatic beta-cell function, as lower insulin levels may suggest that the pancreas is producing insulin more efficiently in response to glucose levels. However, further investigation and clinical assessment would be necessary to fully understand the underlying mechanisms behind these changes.

**Figure 2:**
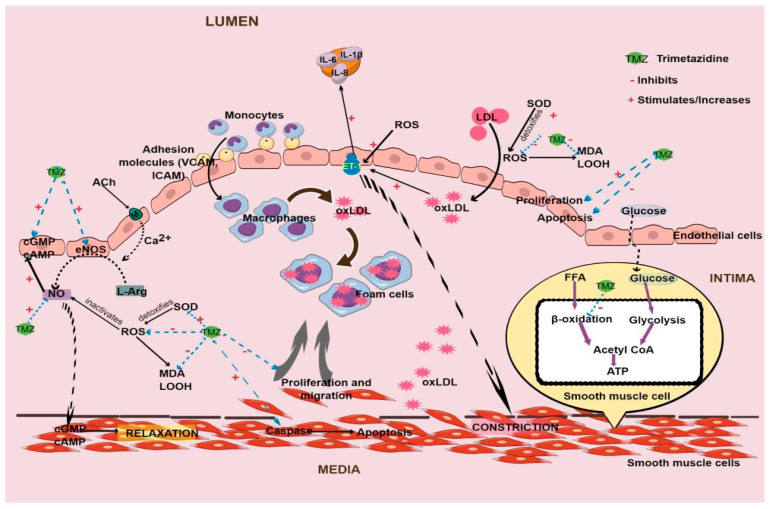
Sites of action of trimetazidine in endothelial dysfunction. ACh, acetylcholine; cAMP, cyclic adenosine monophosphate; cGMP, cyclic guanosine monophosphate; eNOS, endothelial nitric oxide synthase; ET-1, endothelin 1; FFA, free fatty acid; ICAM, intercellular adhesion molecule; IL, interleukin; L-Arg, L-arginine; LDL, low-density lipoprotein; LOOH, lipid hydroperoxides; M, muscarinic receptor; MDA, malondialdehyde; NO, nitric oxide; oxLDL, oxidized low-density lipoprotein; ROS, reactive oxygen species; SOD, superoxide dismutase; VCAM, vascular cell adhesion molecule.

The reduction in fasting blood citrate and β-hydroxybutyrate levels may reflect improved glucose metabolism and reduced reliance on fatty acid oxidation [53]. The findings collectively suggest a metabolic shift indicative of improved glucose metabolism and efficiency (Figure 2). These changes align closely with the known mechanisms of trimetazidine that enhances myocardial energy production by favoring glucose oxidation over fatty acid metabolism, thereby improving myocardial energy efficiency [54]. Consequently, the reduction in reliance on non-glucose substrates for energy production leads to improved glucose metabolism and glycemic control. These metabolic improvements may have significant implications for endothelial function and cardiovascular health. Endothelial dysfunction, characterized by impaired endothelial function and increased inflammation, is a key precursor to cardiovascular diseases such as atherosclerosis. The observed metabolic changes, particularly the reduction in fasting blood glucose, citrate, β-hydroxybutyrate, and insulin levels, are consistent with improvements in endothelial function. Reduced inflammation and oxidative stress, along with improved glucose metabolism, may contribute to the amelioration of endothelial dysfunction. Hyperglycemia is also known to deplete endothelial NO level, leading to impairment of vasorelaxation [55]. Overall, the reported effects of trimetazidine suggest a potential for improving endothelial function and reducing the risk of cardiovascular complications in patients with diabetes mellitus.

The protective effects of trimetazidine were more prominent in patients who performed exercise training. It was believed that combination of the drug and exercise exerted synergistic effects [33] since individually the treatments lowered blood glucose level [33,56]. However, it is worth mentioning that a study by Rehberger-Lizokar and Šebeštjen [41] showed no significant effect of trimetazidine on fasting blood glucose levels in diabetic patients with ischemic heart disease, particularly when compared to a ranolazine-treated group. Notably, this study did not include a placebo-treated group, which might have provided a more comprehensive comparison.

Similar results were observed in animal studies. Administration of trimetazidine at doses of 10 and 30 mg/kg/day led to a reduction in blood glucose levels in diabetic rodents [10,52]. This effect was attributed to a decrease in insulin resistance, as indicated by a decline in the Homeostatic Model Assessment of Insulin Resistance (HOMA-IR) index in diabetic mice fed a high-fat diet. Furthermore, treatment with trimetazidine resulted in elevated levels of phosphorylated Akt (p-Akt) and phosphorylated insulin receptor substrate-1 [52]. In mouse myoblasts exposed to lipid overload, it increased cellular glucose uptake, an effect that was associated with upregulation of p-Akt and phosphorylated insulin receptor substrate-1 [52]. However, trimetazidine had no significant effect on glucose parameters in diabetic rats despite significant beneficial effects on vascular smooth muscle morphology [11].

The significance of the impact of trimetazidine on metabolic parameters, particularly blood glucose levels, in diabetic patients is still a matter of ongoing research and debate. While some studies have reported potential benefits such as improvements in insulin sensitivity and glucose utilization, the overall impact may not be considered significant in all cases. The effects of trimetazidine on blood glucose levels can vary among individuals and may depend on factors such as the severity of diabetes, concurrent medications, and the specific metabolic profile of the patient.

Taken together, it could be summarized that trimetazidine reduces blood glucose levels by decreasing insulin resistance, thereby improving endothelial function. However, studies investigating the effects of trimetazidine on blood glucose metabolism at the molecular level are still lacking. Other aspects of glucose metabolism, such as glucose transporter type 4 (GLUT4) [57] and Twist1, a transcription factor associated with lipid and glucose metabolism [58], could also be of interest.

## 5. Effects of Trimetazidine on Atherosclerosis

Atherosclerosis, starting as a response to arterial endothelium damage, underscores endothelial dysfunction as a primary event in its pathogenesis. As atherosclerosis advances, the accumulation of inflammatory cells, lipids, and fibrous tissues in the arterial intima further exacerbates endothelial dysfunction. Elevated low-density lipoprotein (LDL) levels, a hallmark of hyperlipidemia, significantly contribute to endothelial dysfunction [18]. LDL is susceptible to oxidation, resulting in its transformation into oxLDL, which is known for its proinflammatory and proatherogenic properties. oxLDL disrupts cholesterol efflux, triggering injurious events like endoplasmic reticulum stress and apoptosis, thereby perpetuating endothelial damage [59].

In exploring therapeutic avenues, human studies on the effects of trimetazidine regarding endothelial dysfunction in atherosclerosis are sparse. Nonetheless, animal studies provide insights. Administration of trimetazidine in rodents on a high-fat diet has exhibited promising outcomes in mitigating atherosclerotic lesions (Table 4) [5,12]. Notably, it enhances plaque stability and reduces susceptibility to rupture by diminishing cholesterol clefts and reinforcing fibrous caps. It improves plaque structural integrity by reducing the number of fragmented elastin fibers [12]. Additionally, it demonstrates potential in reducing the ratio of tunica intima to tunica media thickness in diabetic rats with carotid injury [11]. Mechanistically, trimetazidine appears to hinder key events such as proliferation, adhesion, and migration in endothelial cells under conditions of oxidative stress [36] and inflammation [11] (Figure 2). These events are critical in the development and progression of atherosclerotic plaques, suggesting that trimetazidine may potentially alleviate endothelial dysfunction.

Despite LDL’s significant role in the pathogenesis of atherosclerosis [60], trimetazidine surprisingly did not notably affect LDL levels in patients with various cardiovascular diseases and experimental models (Table 5) [5,10,11,33,41,43]. This suggests that trimetazidine may mitigate endothelial dysfunction and the progression of atherosclerosis through alternative mechanisms, such as its antioxidant and anti-inflammatory properties. Additionally, it demonstrated an ability to increase the aortic energy charge, as evidenced by elevated levels of ATP, ADP, and AMP in atherosclerotic aortas. This increase was associated with a reduction in atherosclerotic events [5].

In summary, endothelial dysfunction is a key factor in both the initiation and progression of atherosclerosis. Although trimetazidine holds promise for mitigating atherosclerosis, additional research is necessary to understand its precise impact on endothelial dysfunction and its effectiveness in clinical settings. Given the pivotal role of oxidative stress in atherosclerosis, it is imperative to investigate the potential involvement of ferroptosis, a form of regulated cell death driven by iron-dependent lipid peroxidation [61].

## 6. Effects of Trimetazidine on Oxidative Stress and Inflammation

Atherosclerosis is a chronic inflammatory condition that impacts the vasculature, triggered by oxidative stress, eventually resulting in endothelial dysfunction. In dyslipidemia, LDL penetrates arterial tunica intima and transforms into oxLDL following oxidation by ROS. oxLDL is a significant component in the process of atherosclerosis [62,63]. 

In patients with cardiovascular issues, treatment involving trimetazidine at a total daily dosage of 60–70 mg for 4 to 24 weeks has demonstrated efficacy in reducing oxidative stress, as evidenced by decreased plasma levels of malondialdehyde (MDA) and lipid hydroperoxides, alongside enhancements in total antioxidant status (Table 6) (Figure 2) [34,39]. The antioxidant properties of trimetazidine stem from its ability to inhibit free fatty acid β-oxidation [43], thus mitigating the excessive production of ROS by monocytes, which may otherwise damage the endothelium through LDL oxidation. Studies have demonstrated that trimetazidine reduces ROS expression and levels in rat aortic smooth muscle and endothelial cells [5,11], and these effects correlate with a decrease in oxLDL levels observed in patients with cardiovascular problems [39]. Additionally, the drug’s ability to reduce oxidative stress coincides with increased activity of superoxide dismutase (SOD), an antioxidant enzyme that transforms superoxide anions (a type of ROS) into less reactive byproducts [64], particularly evident in aortic smooth muscle cells subjected to oxLDL or hydrogen peroxide to simulate atherosclerotic conditions [5]. The antioxidant effects of trimetazidine are consistently observed in numerous studies including experimental neurodegeneration [65,66]. Molecular investigations into the antioxidant effects of trimetazidine in endothelial dysfunction are currently lacking. However, it has been reported to activate the nuclear factor erythroid 2-related factor 2 (Nrf2) signaling pathway—a transcription factor pivotal in cellular defense against oxidative stress—in a mouse model of skeletal muscle insulin resistance [52]. Upon activation, Nrf2 translocates to the nucleus, where it binds to antioxidant response elements, consequently regulating the transcription of antioxidant enzymes such as heme oxygenase-1 (HO-1) and NAD(P)H:quinone oxidoreductase 1 (NQO1) [67]. Notably, trimetazidine has been shown to elevate the expression of Nrf2 and both enzymes, as well as enhance SOD activity and reduce MDA content in the aforementioned mouse model [36]. Nrf2 is regulated by Kelch-like ECH-associated protein 1 (Keap1) [67]. However, the precise mechanisms through which trimetazidine influences Nrf2 activation, or whether it directly interacts with Keap1, remain unclear.

oxLDL can activate inflammatory pathways within endothelial cells, leading to the production of inflammatory cytokines and adhesion molecules. This process exacerbates the inflammatory response associated with atherosclerosis. Trimetazidine has been shown to suppress inflammatory events in various animal models of atherosclerosis and diabetes (Table 6) [10,11,12]. Its anti-inflammatory effects have been reported in many studies, including muscle atrophy and mercury-induced nephrotoxicity models [65,66,69] It is believed the drug exerts its anti-inflammatory effect via inhibition of the NOD-like receptor family pyrin domain containing 3/gasdermin D (NLRP3/GSDMD) pathway, AMP-activated protein kinase (AMPK), and galectin-3 [66,69,70].

Propagated inflammation results in the recruitment of immune cells, such as T-lymphocytes and monocytes, to the site of injury. This recruitment is facilitated by adhesion molecules, including vascular cell adhesion molecule (VCAM), intercellular adhesion molecule (ICAM), and E-selectin [71]. However, trimetazidine has demonstrated no significant effect on adhesion molecules in inflamed endothelial cells. Nonetheless, it does reduce the levels of cytokines such as interleukin 1β in stimulated macrophages [12]. These findings suggest that trimetazidine suppresses the release of cytokines from T cells for the conversion of macrophages from monocytes but does not affect the earlier event of cell adhesion.

While trimetazidine predominantly influences oxidative stress over inflammatory events in endothelial cells, its observed effects include a reduction in atherosclerotic plaques [5,12], leading to enhanced endothelial function [12]. However, to gain a comprehensive understanding of its impact on endothelial dysfunction, further investigation into additional mechanistic parameters is warranted. For instance, exploring factors such as Keap-1, which regulates oxidative stress, or the role of various components of the regulatory subunit of the IκB kinase (IKK) complex, namely the α, β, and γ subunits, which are involved in the activation of the inflammatory nuclear factor-kappa B (NF-κB) signaling pathway [72], could provide valuable insights.

## 7. Effects of Trimetazidine on Endothelial Cell and Vascular Smooth Muscle Cell Apoptosis and Angiogenesis

Apoptosis and angiogenesis are pivotal processes intimately involved in endothelial dysfunction. Apoptosis of endothelial cells exacerbates endothelial dysfunction, resulting in compromised endothelial barrier function, disruption of cell–cell junctions, and impaired regulation of vascular permeability and tone [4]. Conversely, angiogenesis serves as a compensatory mechanism to restore tissue perfusion and oxygenation in endothelial dysfunction [73]. This process entails the formation of new blood vessels and is crucial for tissue repair. Apoptosis is involved in vascular remodeling that takes place during angiogenesis [74].

Trimetazidine suppresses neointimal proliferation and cell migration while promoting apoptosis by elevating caspase activity in vascular smooth muscle cells following vascular injury (Table 7) [5,11], suggestive of reduced vascular remodeling. Trimetazidine induces a shift in myocardial energy metabolism from fatty acid oxidation towards glucose oxidation, leading to increased ATP production and improved energy efficiency [50,75]. In vascular smooth muscle cells, this metabolic shift may disrupt the balance of energy substrates required for cell proliferation. Conversely, increased glucose oxidation induced by trimetazidine may promote apoptosis by altering cellular redox status and mitochondrial function [76]. It is also plausible that trimetazidine, by enhancing tissue oxygenation and alleviating ischemic conditions, mitigates the activation of hypoxia-inducible factor (HIF) in vascular smooth muscle cells. These events result in reduced expression of genes associated with proliferation and survival. HIF serves as a transcription factor governing cellular responses to hypoxia, including angiogenesis and cell survival [77].

Trimetazidine can directly or indirectly modulate signaling pathways involved in the proliferation and apoptosis of vascular smooth muscle cells. Studies have demonstrated its ability to activate AMP-activated protein kinase (AMPK), a regulator that inhibits cell proliferation and promotes apoptosis in various cell types, including vascular smooth muscle cells [75,80,81]. Additionally, trimetazidine may influence other signaling pathways such as phosphoinositide-3-kinase/protein kinase B/mammalian target of rapamycin (PI3K/Akt/mTOR) [82] and mitogen-activated protein kinase (MAPK) [83], which are known to regulate cell survival and proliferation. Furthermore, trimetazidine’s anti-inflammatory properties may indirectly impact vascular smooth muscle cell proliferation and survival by suppressing proproliferative and antiapoptotic signals induced by inflammatory cytokines and mediators [84]. This multifaceted modulation of signaling pathways highlights the potential of trimetazidine as a therapeutic agent for regulating vascular smooth muscle cell behavior in various pathological conditions.

In contrast, endothelial cells demonstrate increased proliferation and reduced apoptotic activity, as evidenced by decreased caspase activity following trimetazidine treatment [11,36]. Similar finding of reduced apoptosis has been reported in lipopolysaccharide-stimulated macrophages, serving as a model of endothelial dysfunction [12]. This effect may be mediated by the upregulation of miR-24 and miR-126, microRNAs crucial in vascular development, angiogenesis, apoptosis, and endothelial cell function [10]. These observations suggest that trimetazidine promotes angiogenesis, indicative of active tissue repair processes. 

Research investigating the influence of trimetazidine on angiogenesis in endothelial dysfunction is currently insufficient. Nonetheless, in rats with pressure overload-induced cardiac hypertrophy, trimetazidine has been observed to enhance the expression of vascular endothelial growth factor (VEGF), a crucial angiogenic factor, as well as platelet endothelial cell adhesion molecule-1 (CD31) which facilitates interactions between endothelial cells and other cell types during angiogenesis. Additionally, cell migration and tube formation, indicative of neovascularization, are increased in endothelial cells treated with trimetazidine [78]. These findings suggest that the drug promotes endothelial cell proliferation and angiogenesis. Consequently, trimetazidine facilitates endothelial cell proliferation as part of the neovascularization process, ultimately improving endothelial function. However, in hyperglycemic conditions, the drug alleviates angiogenesis in retinal endothelial cells, as evidenced by reduced cell migration and invasion, decreased tube formation, and VEGF expression [79]. In diabetic retinopathy, characterized by an abnormal increase in angiogenesis, trimetazidine reverses these detrimental effects [79]. The findings of these studies suggest that trimetazidine modulates pathological angiogenesis.

Research indicates that the protective effects of trimetazidine on angiogenesis stem from the activation of Akt and the facilitation of heat shock factor 1 (HSF1) nuclear translocation [78]. HSF1 exerts its influence on angiogenesis by directly modulating the expression of key angiogenic factors, notably VEGF [85]. Evidence suggests that inhibition of HSF1 abolishes the beneficial effects of trimetazidine in endothelial cells [78]. In diabetic retinopathy, trimetazidine modulates angiogenesis by activating the PI3K/Akt/mTOR signaling pathway [79]. This, in turn, suppresses autophagy—a cellular process responsible for breaking down and recycling damaged or unnecessary components within cells, thereby maintaining cellular health and adapting to stress [72]. Evidence of this modulation includes reduced levels of microtubule-associated protein light chain 3 II (LC3-II) and beclin 1, as well as increased expression of p62 [79]. Beclin-1 initiates the autophagy process [86], LC3-II participates in autophagosome formation, and p62 is involved in cellular component degradation through autophagy [72]. Decreased autophagy is linked to diminished apoptosis activity in endothelial cells [87]. In summary, trimetazidine appears to regulate angiogenesis and autophagy, influencing endothelial dysfunction. However, further study is needed to fully understand the role of trimetazidine in autophagy.

Similarly, the precise mechanisms through which trimetazidine influences apoptosis in the endothelium are not fully understood, primarily due to limited research in this area. This gap in knowledge poses a challenge to the potential therapeutic application of trimetazidine in endothelial dysfunction. However, it is hypothesized that trimetazidine may modulate intracellular signaling pathways involved in apoptosis regulation, potentially by activating the Nrf2 signaling pathway [52]. Activation of Nrf2 can lead to the upregulation of antioxidant enzymes and the suppression of apoptotic pathways [88]. Trimetazidine may indirectly inhibit apoptotic signaling pathways activated by oxidative stress, or directly interact with apoptotic proteins involved in the regulation of cell death. It has been suggested that trimetazidine may inhibit the activity of poly ADP-ribose polymerase (PARP), a caspase substrate involved in DNA repair and maintenance. Inhibition of PARP activity could promote apoptosis in certain cellular contexts [89]. Trimetazidine has been reported to regulate the expression of B-cell lymphoma 2 (Bcl-2) family proteins, which play key roles in apoptosis regulation. It has been reported to increase the expression of the antiapoptotic protein Bcl-2 while decreasing the expression of the proapoptotic protein Bcl-2-associated X protein (Bax) in experimental models [90]. Similar effects may occur in the endothelium. Additionally, its metabolic effects [75] and anti-ischemic properties [8] may supply endothelial cells with the necessary resources for proliferation and survival, further mitigating apoptosis. However, further investigation is warranted to fully elucidate the role of trimetazidine in regulating endothelial cell apoptosis and its therapeutic implications for endothelial dysfunction.

Further investigation is warranted to fully elucidate the precise mechanisms by which trimetazidine impacts endothelial cell proliferation and apoptosis. Exploration of endothelial mitochondrial function, including parameters such as the dynamin-related protein 1 (Drp1)-mediated pathway [91], should also be pursued, given the significant role of apoptosis in mitochondrial function. Nevertheless, the angiogenic and antiapoptotic properties of trimetazidine, along with its metabolic effects and ability to alleviate ischemic conditions, likely contribute to its observed effects on endothelial cells. The protective effects observed with trimetazidine therapy underscore its potential as an effective treatment strategy for patients with ischemic heart disease.

## 8. Limitations

While compelling evidence suggests that trimetazidine exhibits protective effects against endothelial dysfunction, a critical factor in maintaining vascular health and preventing cardiovascular diseases, there are several limitations to its therapeutic use in this context. Firstly, it poses a limited direct effect on endothelial function, primarily targeting myocardial metabolism rather than directly influencing endothelial cells. Consequently, its direct effects on endothelial cells are not well established. Furthermore, the specific mechanisms by which trimetazidine may influence endothelial function, involving complex molecular and cellular processes, are not fully understood. 

Trimetazidine therapy may yield inconsistent outcomes due to patient compliance issues. Initially, it is prescribed thrice daily in 20 mg immediate-release tablets. However, to enhance compliance, a modified-release formulation of 35 mg tablets taken twice daily has been suggested [92]. Switching to a slow-release tablet formulation could potentially improve compliance, thus optimizing the therapeutic benefits while minimizing risks. 

Trimetazidine is primarily eliminated unchanged, with over 60% excreted through urine [93]. Consequently, it is contraindicated in patients with chronic kidney disease [19], as its use in such individuals can significantly elevate plasma levels [94]. Additionally, trimetazidine may interact with medications prescribed for other coexisting conditions in patients with endothelial dysfunction, potentially compromising its efficacy or increasing the likelihood of adverse effects. Recent findings suggest that trimetazidine can attenuate the effects of antiepileptic drugs such as phenytoin and carbamazepine without affecting their brain levels [95]. Therefore, healthcare providers must exercise caution and meticulously assess potential drug interactions when prescribing trimetazidine to patients with endothelial dysfunction.

## 9. Conclusions and Avenues for Future Research

As of now, the mechanisms by which trimetazidine alleviates endothelial dysfunction likely involve the regulation of angiogenesis and apoptosis, the reduction in oxidative stress and inflammation, and the augmentation of endothelial NO bioavailability. However, further studies are essential to fully elucidate the precise molecular pathways involved and to explore potential long-term effects. Investigating potential synergistic effects with other cardiovascular medications could offer valuable insights into maximizing its therapeutic benefits. Moreover, examining the interplay between trimetazidine and lifestyle interventions, such as dietary modifications, in enhancing endothelial function warrants further investigation.

While ongoing research on trimetazidine and its effects on endothelial function holds promise for advancing cardiovascular medicine and enhancing patient outcomes, it is important to note that no clinical trials have been conducted to explore the long-term benefits of the drug specifically on endothelial dysfunction. Currently, its usage is mainly for symptomatic control of angina and is typically discontinued once treatment goals are met. To confirm its positive effects on endothelial dysfunction, long-term administration of trimetazidine is necessary to assess whether it could prevent the further initiation of atheroma after improving endothelial dysfunction. Additionally, future studies should consider the translational potential of trimetazidine in addressing endothelial dysfunction-related complications beyond cardiovascular diseases, including diabetic nephropathy, erectile dysfunction, and pre-eclampsia.

## Figures and Tables

**Table 1:** Effects of trimetazidine on endothelium-dependent vasodilation and hemodynamic parameters in patients with cardiovascular disease and experimental animals.

Subjects	Dose of Trimetazidine	Type of Study	Findings	Reference
Patients with CHD and primary hypertension (*n* = 68)	Combined with berberine. No information on the dose and duration of treatment		↑ FMD	[9]
Alloxan-induced diabetic rats	10 and 30 mg/kg (p.o.) for 8 weeks		Both doses: ↑ systolic pressure	[10]
Patients with IHD and LV dysfunction (*n* = 116)	20 mg (p.o., t.i.d.) for 8 weeks	Randomized longitudinal controlled	Without exercise (vs. control): ↑ % EDD ↔ systolic BP ↔ diastolic BP With exercise (vs. TMZ): ↑ % EDD ↑ systolic BP ↑ diastolic BP	[33]
Patients with CHF secondary to ischemic cardiomyopathy (*n* = 51)	20 mg (p.o., t.i.d.) for 4 weeks	Randomized	↑ ACh-induced vasorelaxation ↔ GTN-induced vasorelaxation ↑ radial artery diameter	[34]
Patients with endothelial dysfunction after sheath injury of radial artery and PCI (*n* = 120)	20 mg (p.o., t.i.d.) for 10 weeks	Randomized	10 weeks after angiography: ↑ radial artery diameter ↑ FMD	[35]
Patients with lower extremity arteriosclerosis obliterans (*n* = 132)	30 mg (p.o., t.i.d.) combined with alprostadil (0.1 µg/day, i.v.) for 14 days	Retrospective	Compared to baseline: ↓ VPV of superficial femoral artery ↓ VPV of posterior tibial artery ↓ VPV of dorsalis pedis artery ↑ blood flow of superficial femoral artery ↑ blood flow of posterior tibial artery ↑ blood flow of dorsalis pedis artery ↑ left ankle brachial index	[37]
Patients with CAD (*n* = 570)	35 mg (XR, p.o., b.i.d.) for 5 years	Randomized	At 6 months: vs. NSTE-ACS: ↓ FMV ↓ vWF activity vs. CCS: ↔ FMV ↔ vWF activity	[39]
Patients with endothelial dysfunction after sheath injury of radial artery (*n* = 60)	20 mg (p.o., t.i.d.) for 10 weeks	Randomized	10 weeks after angiography: ↑ radial artery diameter ↑ FMD ↑ NMD	[40]
Patients with ischemic heart disease (*n* = 56)	35 mg (p.o. bi.d.) for 12 weeks	Randomized	↔ GTN-induced diameter ↔ GTN-induced blood flow ↑ % GTN-induced blood flow ↑ % FMD ↑ FMD/GTN-induced blood flow ↑ hyperemia-induced arterial diameter ↔ hyperemia-induced blood flow ↑ baseline diameter ↑ baseline blood flow	[41]

ACh, acetylcholine; ACS, acute coronary syndrome; AT, anaerobic threshold; b.i.d., twice daily; BP, blood pressure; CAD, coronary artery disease; CHD, coronary heart disease; CHF, congestive cardiac failure; CCS, chronic coronary syndrome; EDD, endothelium-dependent dilation; FMD, flow-mediated dilation; FMV, flow-mediated vasodilation; GTN, glyceryl trinitrate; LV, left ventricle; NMD, nitrate-mediated dilation; NSTE, non-ST elevation; PCI, percutaneous coronary intervention; p.o., per oral; t.i.d., three times daily; TMZ, trimetazidine; VPC, vascular peak velocity; VPV, vascular peak velocity; vWF, von Willebrand factor; XR, extended release; ↑, increase; ↓, decrease; ↔, no change.

**Table 2:** Effects of trimetazidine on vasoactive substances in patients with cardiovascular disease and experimental study.

Subjects	Dose of Trimetazidine	Type of Study	Findings	Reference
Patients with CHD and primary hypertension (*n* = 68)	Combined with berberine. No information on the dose and duration of treatment		↑ plasma NO ↑ eNOS mRNA	[9]
H_2_O_2_-induced HUVECs	10 µM		↑ eNOS protein ↑ eNOS mRNA ↑ NO	[36]
Patients with lower extremity arteriosclerosis obliterans (*n* = 132)	30 mg (p.o., t.i.d.) combined with alprostadil (0.1 µg/day, i.v.) for 14 days	Retrospective	Compared to baseline: ↔ serum NO	[37]
Patients with unstable angina in perioperative PCI (*n* = 97)	20 mg (p.o., t.i.d.,) 24 h before and after PCI	Randomized	↔ serum NO	[38]
Patients with T2DM and hypokinetic cardiomyopathy secondary to IHD (*n* = 16)	20 mg (p.o., tid) for 30 days and 12 months	Randomized, double-blind, crossover study	Short-term: ↓ plasma ET-1 Long-term: ↓ plasma ET-1	[42]
Patients with T2DM and ischemic cardiomyopathy (*n* = 15)	20 mg (p.o., tid) for 15 days	Randomized, double-blind, placebo-controlled, crossover parallel study	With euglycemic clamp: ↓ endothelial ET-1 release ↓ endothelial ET-1 ↑ endothelial cGMP ↑ endothelial cGMP release	[43]

cGMP, cyclic guanosine monophosphate; CHD, coronary heart disease; eNOS, endothelial nitric oxide synthase; ET-1, endothelin-1; HUVECs, human umbilical vein endothelial cells; IHD, ischemic heart disease; NO, nitric oxide; PCI, percutaneous coronary intervention; p.o., per oral; T2DM, type 2 diabetes mellitus; t.i.d., three times daily; ↑, increase; ↓, decrease; ↔, no change.

**Table 3:** Effects of trimetazidine on blood glucose metabolism in patients with diabetes and cardiac disease and experimental diabetic animals.

Subjects	Dose of Trimetazidine	Type of Study	Findings	Reference
Alloxan-induced diabetic rats	10 and 30 mg/kg (p.o.) for 8 weeks		Both doses: ↓ fasting blood glucose	[10]
Carotid injury in diabetic rats	10 and 20 mg/kg (p.o.) for 2 weeks before and after injury		↔ fasting glucose ↔ post-load glucose ↔ fasting insulin ↔ HOMA-IR ↓ adiponectin	[11]
Patients with IHD and LV dysfunction (*n* = 116)	20 mg (p.o., t.i.d.) for 8 weeks	Randomized longitudinal controlled	Without exercise (vs. control): ↓ glucose ↔ HbA1C With exercise (vs. TMZ): ↓ glucose ↓ HbA1C	[33]
Patients with IHD (*n* = 56)	35 mg (p.o. b.i.d.) for 12 weeks	Randomized	↔ fasting blood glucose (compared to ranolazine-treated group)	[41]
Patients with T2DM and hypokinetic cardiomyopathy secondary to IHD (*n* = 16)	20 mg (p.o., t.i.d.) for 30 days and 12 months	Randomized, double-blind, crossover study	Short-term: ↓ fasting blood glucose ↑ end-clamp forearm glucose uptake ↔ HbA1C ↑ M value Long-term: ↔ fasting blood glucose ↑ end-clamp FGU ↔ HbA1C ↑ M value	[42]
Patients with T2DM and ischemic cardiomyopathy (*n* = 15)	20 mg (p.o., t.i.d.) for 15 days	Randomized, double-blind, placebo-controlled, crossover parallel study	↑ FGU ↑ forearm glucose oxidation ↔ forearm glucose storage ↓ basal forearm non-glucose glycolysis ↓ fasting blood glucose ↓ fasting blood citrate ↓ fasting blood β-OH-butyrate ↓ fasting insulin	[43]
High-fat diet mouse insulin resistance model	10 mg/kg (p.o.) for 8 weeks		↓ fasting blood glucose ↓ plasma insulin ↓ HOMA-IR	[52]

HbA1C, hemoglobin A1c; FGU, forearm glucose uptake; HOMA-IR, Homeostatic Model Assessment of Insulin Resistance; IHD, ischemic heart disease; LV, left ventricle; M value, index of total body glucose disposal; β-OH-butyrate, β-hydroxybutyrate; p.o., per oral; T2DM, type 2 diabetes mellitus; t.i.d., three times daily; TMZ, trimetazidine; ↑, increase; ↓, decrease; ↔, no change.

**Table 4:** Effects of trimetazidine on atherosclerotic plaque in experimental animals.

Models	Dose of Trimetazidine	Findings	Reference
High fat/vitamin D-induced atherosclerosis in rats	30 mg/kg/day (p.o.) for 12 weeks	↓ atherosclerotic lesion	[5]
Carotid injury in diabetic rats	10 and 20 mg/kg (p.o.) 2 weeks before and after injury	↓ intima–media ratio	[11]
Rat aortic SMCs exposed to TNF-α	25–500 µM	100–500 µM: ↓ cell proliferation 500 µM: ↓ cell migration	[11]
High-fat-diet-induced atherosclerosis in LDL-R^−/−^ male mice	15 mg/kg/day via tap water for 8 weeks (post-treatment)	↓ atherosclerotic plaque area ↓ cholesterol clefts ↑ fibrous cap ↓ elastin fiber breaks	[12]
H_2_O_2_-induced HUVECs	10 µM	↓ cell migration ↓ cell adhesion	[36]

HUVECs, human umbilical vein endothelial cells; LDL-R^−/−^, low-density lipoprotein receptor knockout model; p.o., per oral; SMCs, smooth muscle cells; TNF-α, tumor necrosis factor α; ↑, increase; ↓, decrease.

**Table 5:** Effects of trimetazidine on blood lipid profile in patients with cardiovascular diseases and animal studies.

Subjects	Dose of Trimetazidine	Type of Study	Findings	Reference
High fat/vitamin D-induced atherosclerosis in rats	30 mg/kg/day (p.o.) for 12 weeks		↔ TG ↔ TC ↔ LDL	[5]
Alloxan-induced diabetic rats	10 and 30 mg/kg (p.o.) for 8 weeks		Both doses: ↓ TC ↓ TG ↔ HDL ↔ LDL ↓ VLDL	[10]
Carotid injury in diabetic rats	10 and 20 mg/kg (p.o.) for 2 weeks before and after injury		↔ TG ↔ HDL ↔ TC ↔ LDL	[11]
Patients with IHD and LV dysfunction (*n* = 116)	20 mg (p.o., t.i.d.) for 8 weeks	Randomized longitudinal controlled	Without exercise (vs. control): ↔ HDL ↔ TG With exercise (vs. TMZ): ↑ HDL ↑ TG	[33]
Patients with IHD (*n* = 56)	35 mg (p.o. b.i.d.) for 12 weeks	Randomized	↔ HDL ↔ LDL ↔ TC ↑ TG	[41]
Patients with T2DM and ischemic cardiomyopathy (*n* = 15)	20 mg (p.o., t.i.d.) for 15 days	Randomized, double-blind, placebo-controlled, crossover parallel study	↔ HDL ↔ FFA ↔ TG ↔ TC ↔ lactate	[43]

FFA, free fatty acid; HDL, high-density lipoprotein; IHD, ischemic heart disease; LDL, low-density lipoprotein; LV, left ventricle; M value, glucose infusion rate during clamp; p.o., per oral; T2DM, type 2 diabetes mellitus; TC, total cholesterol; TG, triglyceride; t.i.d., three times daily; ↑, increase; ↓, decrease; ↔, no change.

**Table 6:** Effects of trimetazidine on oxidative stress and inflammation in patients with cardiovascular disease and animal studies.

Subjects	Dose of Trimetazidine	Type of Study	Findings	Reference
High fat/vitamin D-induced atherosclerosis in rats	30 mg/kg/day (p.o.) for 12 weeks		↓ aortic ROS	[5]
oxLDL-induced proliferation in human aortic smooth muscle cells	10 µM		↓ ROS ↑ SOD activity ↓ MDA	[5]
H_2_O_2_-induced proliferation in human aortic smooth muscle cells	10 µM		↓ ROS ↑ SOD activity ↓ MDA	[5]
Alloxan-induced diabetic rats	10 and 30 mg/kg (p.o.) for 8 weeks		Both doses: ↓ plasma MDA ↓ plasma TNFα ↓ plasma IL-6	[10]
Carotid injury in diabetic rats	10 and 20 mg/kg (p.o.) for 2 weeks before and after injury		↓ intimal 8-OH-dG ↓ intimal hsCRP ↓ intimal TNFα	[11]
Rat aortic smooth muscle cells and HUVECs exposed to lysophosphatidylcholine	250 and 500 µM		↓ ROS expression	[11]
Rh-IL-1β-stimulated HUVECs	500 µM (pre-treatment)		↔ adherent granulocytes ↔ E-selectin ↔ ICAM-1 ↔ VCAM-1 ↔ IL-8 ↔ nuclear p65	[12]
LPS/nigericin-induced macrophages	500 µM (pre-treatment)		↓ IL-1β ↓ M1 IL-1β ↔ CD80 mRNA ↔ TNF mRNA ↔ uPAR mRNA	[12]
High-fat-diet-induced atherosclerosis in LDL-R^−/−^ male mice	15 mg/kg/day via tap water for 8 weeks (post-treatment)		↓ plaque cleaved IL-1β area ↓ plaque neutrophil extracellular traps ↓ serum IL-1β ↓ serum IL-18	[12]
Patients with CHF secondary to ischemic cardiomyopathy (*n* = 51)	20 mg (p.o., t.i.d.) for 4 weeks	Randomized	↓ plasma MDA ↓ plasma LOOH	[34]
H_2_O_2_-induced HUVECs	10 µM		↓ MDA ↑ SOD	[36]
Patients with lower extremity arteriosclerosis obliterans (*n* = 132)	30 mg (p.o., t.i.d.) combined with alprostadil (0.1 µg/day, i.v.) for 14 days	Retrospective	Compared to plasmin+alprostadil: ↔ hs-CRP	[37]
Patients with CAD (*n* = 570)	35 mg (XR, p.o., b.i.d.) for 6 months	Randomized	vs. NSTE-ACS ↑ TAS ↓ oxLDL ↔ MPO ↓ CRP ↓ fibrinogen vs. CCS: ↑ TAS ↔ oxLDL ↔ MPO ↔ CRP ↔ fibrinogen	[39]
Femoral artery ligation in diabetic mice	10 mg/kg/day (i.g.) for 2 weeks (post-treatment)		↓ serum ICAM-1	[68]

b.i.d., twice daily; CAD, coronary artery disease; CCS, chronic coronary syndrome; CHF, congestive heart failure; CRP, C-reactive protein; hs-CRP, high-sensitivity C-reactive protein; HUVECs, human umbilical vein endothelial cells; ICAM-1, intercellular adhesion molecule-1; IL-1β, interleukin 1β; IL-6, interleukin 6; IL-8, interleukin 8; i.v., intravenous; LDL-R^−/−^, low-density lipoprotein receptor knockout model; LPS, lipopolysaccharide; LOOH, M1, proinflammatory macrophages; MDA, malondialdehyde; MPO, myeloperoxidase; NSTE-ACS, non-ST elevation acute coronary syndrome; 8-OH-dG, 8-hydroxy-2′-deoxyguanosine; oxLDL, oxidized low-density lipoprotein; Rh-IL-1β, recombinant human interleukin 1β; ROS, reactive oxygen species; SOD, superoxide dismutase; TAS, total antioxidant status; t.i.d., three times daily; TNF, tumor necrosis factor; VCAM, vascular cell adhesion molecule; XR, extended release; ↑, increase; ↓, decrease; ↔, no change.

**Table 7:** Effects of trimetazidine on endothelial cells and vascular smooth muscle cell apoptosis and angiogenesis in experimental studies.

Models	Dose of Trimetazidine	Findings	Reference
oxLDL-induced proliferation in human aortic smooth muscle cells	0.1, 1, and 10 µM	↓ proliferation	[5]
Alloxan-induced diabetic rats	10 and 30 mg/kg (p.o.) for 8 weeks	Both doses: ↑ plasma miR24 protein ↑ plasma miR126 protein	[10]
Carotid injury in diabetic rats	10 and 20 mg/kg (p.o.) 2 weeks before and after injury	↓ muscle proliferation index	[11]
Rat aortic smooth muscle cells exposed to TNFα	50, 100, 250, and 500 µM	250 and 500 µM: ↓ proliferation All concentrations: ↑ caspase activity 500 µM: ↓ migration	[11]
Rat aortic SMCs exposed to TNF-α	25–500 µM	↑ viability ↓ active caspase-3 ↓ apoptotic cells	[11]
LPS/nigericin-induced macrophages	500 µM (pre-treatment)	↓ cleaved caspase-1	[12]
High-fat-diet-induced atherosclerosis in LDL-R^−/−^ male mice	15 mg/kg/day via tap water for 8 weeks (post-treatment)	↓ aortic plaque cleaved caspase-1 ↓ aortic plaque cell count	[12]
H_2_O_2_-induced HUVECs	10 µM	↓ apoptosis ↑ proliferation	[36]
TAC-induced cardiac hypertrophy mice	2.8 mg/100 µL (i.g.)	↑ CD31 ↑ VEGF	[78]
PE-induced HUVECs	5 µM	↑ proliferation ↑ cell migration ↑ tube formation	[78]
Diabetic retinopathy in human retinal endothelial cells	1–10 µM	↓ proliferation ↓ cell migration ↓ VEGF ↓ cell invasion ↓ tube formation ↓ cell permeability	[79]

CD31, platelet endothelial cell adhesion molecule-1; HUVECs, human umbilical vein endothelial cells; i.g., intragastric; LDL-R^−/−^, low-density lipoprotein receptor knockout model; LPS, lipopolysaccharide; oxLDL, oxidized low-density lipoprotein; PE, phenylephrine; TAC, transverse aortic constriction; TNFα, tumor necrosis factor α; p.o., per oral; VEGF, vascular endothelial growth factor; ↑, increase; ↓, decrease.

## Data Availability

Data can be shared upon request.

## References

[1] WHO (2023). Cardiovascular Diseases. https://www.who.int/health-topics/cardiovascular-diseases#tab=tab_1.

[2] Fan J., Watanabe T. (2022). Atherosclerosis: Known and unknown. Pathol. Int..

[3] Clyne A.M. (2021). Endothelial response to glucose: Dysfunction, metabolism, and transport. Biochem. Soc. Trans..

[4] Cheng H., Di G., Gao C.C., He G., Wang X., Han Y.L., Sun L.A., Zhou M.L., Jiang X. (2021). FTY720 reduces endothelial cell apoptosis and remodels neurovascular unit after experimental traumatic brain injury. Int. J. Med. Sci..

[5] Zheng S., Du Y., Peng Q., Fan X., Li J., Chen M. (2018). Trimetazidine protects against atherosclerosis by changing energy charge and oxidative stress. Med. Sci. Monit..

[6] Hellenthal K.E.M., Brabenec L., Wagner N.M. (2022). Regulation and dysregulation of endothelial permeability during systemic inflammation. Cells.

[7] Bubnova M.G., Aronov D.M. (2021). Efficacy of trimetazidine—An inhibitor of free fatty acids oxidation in the treatment of patients with stable angina pectoris and heart failure. Kardiologiia.

[8] He C., Cao S., Tong Z., Wang W., Zhang Y., Guo C. (2018). Trimetazidine ameliorates myocardial ischemia-reperfusion injury. Pak. J. Pharm. Sci..

[9] Zhang H., Niu H., Yuan X., Chang J., Wang X. (2018). Trimetazidine combined with berberine on endothelial function of patients with coronary heart disease combined with primary hypertension. Exp. Ther. Med..

[10] Ramezani-Aliakbari F., Badavi M., Dianat M., Mard S.A., Ahangarpour A. (2020). Trimetazidine increases plasma microRNA-24 and microRNA-126 levels and improves dyslipidemia, inflammation and hypotension in diabetic rats. Iran J. Pharm. Res..

[11] Yoon J.W., Cho B.J., Park H.S., Kang S.M., Choi S.H., Jang H.C., Shin H., Lee M.J., Kim Y.B., Park K.S. (2013). Differential effects of trimetazidine on vascular smooth muscle cell and endothelial cell in response to carotid artery balloon injury in diabetic rats. Int. J. Cardiol..

[12] Hohensinner P.J., Lenz M., Haider P., Mayer J., Richter M., Kaun C., Goederle L., Brekalo M., Salzmann M., Sharma S. (2021). Pharmacological inhibition of fatty acid oxidation reduces atherosclerosis progression by suppression of macrophage NLRP3 inflammasome activation. Biochem. Pharmacol..

[13] Chrusciel P., Rysz J., Banach M. (2014). Defining the role of trimetazidine in the treatment of cardiovascular disorders: Some insights on its role in heart failure and peripheral artery disease. Drugs.

[14] Kwon J., Yu Y.M., Kim S., Jeong K.H., Lee E. (2019). Association between trimetazidine and parkinsonism: A population-based study. Neuroepidemiology.

[15] Pintér D., Kovács M., Harmat M., Juhász A., Janszky J., Kovács N. (2019). Trimetazidine and parkinsonism: A prospective study. Park. Relat. Disord..

[16] Ferrari R., Ford I., Fox K., Challeton J.P., Correges A., Tendera M., Widimský P., Danchin N., ATPCI investigators (2020). Efficacy and safety of trimetazidine after percutaneous coronary intervention (ATPCI): A randomised, double-blind, placebo-controlled trial. Lancet.

[17] Oesterle A., Laufs U., Liao J.K. (2017). Pleiotropic effects of statins on the cardiovascular system. Circ. Res..

[18] Xu S., Ilyas I., Little P.J., Li H., Kamato D., Zheng X., Luo S., Li Z., Liu P., Han J. (2021). Endothelial dysfunction in atherosclerotic cardiovascular diseases and beyond: From mechanism to pharmacotherapies. Pharmacol. Rev..

[19] Ferrari R., Camici P.G., Crea F., Danchin N., Fox K., Maggioni A.P., Manolis A.J., Marzilli M., Rosano G.M.C., Lopez-Sendon J.L. (2018). Expert consensus document: A ‘diamond’ approach to personalized treatment of angina. Nat. Rev. Cardiol..

[20] Berkels R., Taubert D., Rosenkranz A., Rösen R. (2003). Vascular protective effects of dihydropyridine calcium antagonists. Involvement of endothelial nitric oxide. Pharmacology.

[21] Ruszkowski P., Masajtis-Zagajewska A., Nowicki M. (2019). Effects of combined statin and ACE inhibitor therapy on endothelial function and blood pressure in essential hypertension—A randomised double-blind, placebo-controlled crossover study. J. Renin Angiotensin Aldosterone Syst..

[22] Oliveira A.C.D., Arismendi M.I., Machado L.S.G., Sato E.I. (2022). Ramipril improves endothelial function and increases the number of endothelial progenitor cells in patients with systemic lupus erythematosus. J. Clin. Rheumatol..

[23] Ames M.K., Atkins C.E., Pitt B. (2019). The renin-angiotensin-aldosterone system and its suppression. J. Vet. Intern. Med..

[24] Oparil S., Williams D., Chrysant S.G., Marbury T.C., Neutel J. (2001). Comparative efficacy of olmesartan, losartan, valsartan, and irbesartan in the control of essential hypertension. J. Clin. Hypertens..

[25] Willenheimer R., Helmers C., Pantev E., Rydberg E., Löfdahl P., Gordon A., Heart Failure Valsartan Exercise Capacity Evaluation Study Group (2002). Safety and efficacy of valsartan versus enalapril in heart failure patients. Int. J. Cardiol..

[26] Medina-Leyte D.J., Zepeda-García O., Domínguez-Pérez M., González-Garrido A., Villarreal-Molina T., Jacobo-Albavera L. (2021). Endothelial dysfunction, inflammation and coronary artery disease: Potential biomarkers and promising therapeutical approaches. Int. J. Mol. Sci..

[27] Cooper S., Teoh H., Campeau M.A., Verma S., Leask R.L. (2019). Empagliflozin restores the integrity of the endothelial glycocalyx in vitro. Mol. Cell. Biochem..

[28] Aini K., Fukuda D., Tanaka K., Higashikuni Y., Hirata Y., Yagi S., Kusunose K., Yamada H., Soeki T., Sata M. (2019). Vildagliptin, a DPP-4 Inhibitor, attenuates endothelial dysfunction and atherogenesis in nondiabetic apolipoprotein E-deficient mice. Int. Heart J..

[29] Zoupa E., Pitsikas N. (2021). The nitric oxide (NO) donor sodium nitroprusside (SNP) and its potential for the schizophrenia therapy: Lights and shadows. Molecules.

[30] Münzel T., Daiber A. (2018). Inorganic nitrite and nitrate in cardiovascular therapy: A better alternative to organic nitrates as nitric oxide donors?. Vascul. Pharmacol..

[31] Kamisah Y., Zuhair J.S.F., Juliana A.H., Jaarin K. (2017). *Parkia speciosa* empty pod prevents hypertension and cardiac damage in rats given N(G)-nitro-l-arginine methyl ester. Biomed. Pharmacother..

[32] Khodabakhsh P., Asgari Taei A., Mohseni M., Bahrami Zanjanbar D., Khalili H., Masoumi K., Haji Abbas Shirazi A., Dargahi L. (2021). Vasoactive peptides: Role in COVID-19 pathogenesis and potential use as biomarkers and therapeutic targets. Arch. Med. Res..

[33] Belardinelli R., Lacalaprice F., Faccenda E., Volpe L. (2008). Trimetazidine potentiates the effects of exercise training in patients with ischemic cardiomyopathy referred for cardiac rehabilitation. Eur. J. Cardiovasc. Prev. Rehabil..

[34] Belardinelli R., Solenghi M., Volpe L., Purcaro A. (2007). Trimetazidine improves endothelial dysfunction in chronic heart failure: An antioxidant effect. Eur. Heart J..

[35] Park K.H., Park D.W., Kim M.K., Kim H.S., Park W.J., Cho G.Y., Choi Y.J. (2012). Effects of sheath injury and trimetazidine on endothelial dysfunction of radial artery after transradial catheterization. J. Interv. Cardiol..

[36] Wu Q., Qi B., Liu Y., Cheng B., Liu L., Li Y., Wang Q. (2013). Mechanisms underlying protective effects of trimetazidine on endothelial progenitor cells biological functions against H_2_O_2_-induced injury: Involvement of antioxidation and Akt/eNOS signaling pathways. Eur. J. Pharmacol..

[37] Yong J., Wang Y., Xing S., Bi Y., Li N., Zhao S. (2019). Efficacy of trimetazidine and plasmin combined with alprostadil in treatment of lower extremity arteriosclerosis obliterans. Exp. Ther. Med..

[38] Shao S., Shi Z., Tse G., Wang X., Ni Y., Liu H., Liu T., Li G. (2019). Effects of trimetazidine pretreatment on endothelial dysfunction and myocardial injury in unstable angina patients undergoing percutaneous coronary intervention. Cardiol. Res. Pract..

[39] Bobescu E., Marceanu L.G., Dima L., Balan A.l, Strempel C.G., Covaciu A. (2021). Trimetazidine therapy in coronary artery disease: The impact on oxidative stress, inflammation, endothelial dysfunction, and long-term prognosis. Am. J. Ther..

[40] Park K.H., Park W.J., Kim M.K., Park D.W., Park J.H., Kim H.S., Cho G.Y. (2010). Effects of trimetazidine on endothelial dysfunction after sheath injury of radial artery. Am. J. Cardiol..

[41] Rehberger-Likozar A., Šebeštjen M. (2015). Influence of trimetazidine and ranolazine on endothelial function in patients with ischemic heart disease. Coron. Artery Dis..

[42] Fragasso G., Piatti P.M., Monti L., Palloshi A., Setola E., Puccetti P., Calori G., Lopaschuk G.D., Margonato A. (2003). Short- and long-term beneficial effects of trimetazidine in patients with diabetes and ischemic cardiomyopathy. Am. Heart J..

[43] Monti L.D., Setola E., Fragasso G., Camisasca R.P., Lucotti P., Galluccio E., Origgi A., Margonato A., Piatti P. (2006). Metabolic and endothelial effects of trimetazidine on forearm skeletal muscle in patients with type 2 diabetes and ischemic cardiomyopathy. Am. J. Physiol. Endocrinol. Metab..

[44] Straub A.C., Beuve A. (2021). A primer for measuring cGMP signaling and cGMP-mediated vascular relaxation. Nitric Oxide.

[45] Feng X., Sureda A., Jafari S., Memariani Z., Tewari D., Annunziata G., Barrea L., Hassan S.T.S., Šmejkal K., Malaník M. (2019). Berberine in cardiovascular and metabolic diseases: From mechanisms to therapeutics. Theranostics.

[46] Siti H.N., Jalil J., Asmadi A.Y., Kamisah Y. (2021). Rutin modulates MAPK pathway differently from quercetin in angiotensin II-induced H9c2 cardiomyocyte hypertrophy. Int. J. Mol. Sci..

[47] Shi Y., Vanhoutte P.M. (2017). Macro- and microvascular endothelial dysfunction in diabetes. J. Diabetes.

[48] Garg S.S., Gupta J. (2022). Polyol pathway and redox balance in diabetes. Pharmacol. Res..

[49] Ciulla T.A., Amador A.G., Zinman B. (2003). Diabetic retinopathy and diabetic macular edema: Pathophysiology, screening, and novel therapies. Diabetes Care.

[50] Lopaschuk G.D., Barr R., Thomas P.D., Dyck J.R. (2003). Beneficial effects of trimetazidine in ex vivo working ischemic hearts are due to a stimulation of glucose oxidation secondary to inhibition of long-chain 3-ketoacyl coenzyme a thiolase. Circ. Res..

[51] Mitrou P., Petsiou E., Papakonstantinou E., Maratou E., Lambadiari V., Dimitriadis P., Spanoudi F., Raptis S.A., Dimitriadis G. (2015). Vinegar consumption increases insulin-stimulated glucose uptake by the forearm muscle in humans with type 2 diabetes. J. Diabetes Res..

[52] Zhang W., Dun Y., You B., Qiu L., Ripley-Gonzalez J.W., Cheng J., Fu S., Li C., Liu S. (2022). Trimetazidine and exercise offer analogous improvements to the skeletal muscle insulin resistance of mice through Nrf2 signaling. BMJ Open Diabetes Res. Care.

[53] Luukkonen P.K., Dufour S., Lyu K., Zhang X.M., Hakkarainen A., Lehtimäki T.E., Cline G.W., Petersen K.F., Shulman G.I., Yki-Järvinen H. (2020). Effect of a ketogenic diet on hepatic steatosis and hepatic mitochondrial metabolism in nonalcoholic fatty liver disease. Proc. Natl. Acad. Sci. USA.

[54] Klonoff D.C., Xu N.Y., Nguyen K.T., Kerr D., Mehta C., Umpierrez G.E., Brooks G.A. (2022). Trimetazidine blocks lipid oxidation-should it be repurposed for prevention and treatment of diabetic ketoacidosis?. J. Diabetes Sci. Technol..

[55] Hoshiyama M., Li B., Yao J., Harada T., Morioka T., Oite T. (2003). Effect of high glucose on nitric oxide production and endothelial nitric oxide synthase protein expression in human glomerular endothelial cells. Nephron. Exp. Nephrol..

[56] Xie Y., Zhao H., Zhao M., Huang H., Liu C., Huang F., Wu J. (2022). Effects of resistance exercise on blood glucose level and pregnancy outcome in patients with gestational diabetes mellitus: A randomized controlled trial. BMJ Open Diabetes Res. Care.

[57] Huang D.D., Shi G., Jiang Y., Yao C., Zhu C. (2020). A review on the potential of resveratrol in prevention and therapy of diabetes and diabetic complications. Biomed. Pharmacother..

[58] Huang L., Xing Y., Ning X., Yu Z., Bai X., Liu L., Sun S. (2023). Roles of Twist1 in lipid and glucose metabolism. Cell. Commun. Signal..

[59] Nasoni M.G., Crinelli R., Iuliano L., Luchetti F. (2023). When nitrosative stress hits the endoplasmic reticulum: Possible implications in oxLDL/oxysterols-induced endothelial dysfunction. Free Radic. Biol. Med..

[60] Kattoor A.J., Kanuri S.H., Mehta J.L. (2019). Role of ox-LDL and LOX-1 in atherogenesis. Curr. Med. Chem..

[61] Bai T., Li M., Liu Y., Qiao Z., Wang Z. (2020). Inhibition of ferroptosis alleviates atherosclerosis through attenuating lipid peroxidation and endothelial dysfunction in mouse aortic endothelial cell. Free Radic. Biol. Med..

[62] Libby P., Buring J.E., Badimon L., Hansson G.K., Deanfield J., Bittencourt M.S., Tokgözoğlu L., Lewis E.F. (2019). Atherosclerosis. Nat. Rev. Dis. Primers.

[63] Libby P. (2021). The changing landscape of atherosclerosis. Nature.

[64] Siti H.N., Jalil J., Asmadi A.Y., Kamisah Y. (2021). *Parkia speciosa* Hassk. Empty pod extract alleviates angiotensin II-induced cardiomyocyte hypertrophy in H9c2 cells by modulating the Ang II/ROS/NO axis and MAPK pathway. Front. Pharmacol..

[65] Sedky A., Famurewa A.C. (2023). Anti-ischemic drug trimetazidine blocks mercury nephrotoxicity by suppressing renal redox imbalance, inflammatory stress and caspase-dependent apoptosis in rats. Drug Chem. Toxicol..

[66] Hassan F.E., Aboulhoda B.E., Ali I.H., Elwi H.M., Matter L.M., Abdallah H.A., Khalifa M.M., Selmy A., Alghamdi M.A., Morsy S.A. (2023). Evaluating the protective role of trimetazidine versus nano-trimetazidine in amelioration of bilateral renal ischemia/reperfusion induced neuro-degeneration: Implications of ERK1/2, JNK and Galectin-3/NF-kappaB/TNF-alpha/HMGB-1 signaling. Tissue Cell.

[67] Syamsunarno M.R.A., Safitri R., Kamisah Y. (2021). Protective effects of *Caesalpinia sappan* Linn. and its bioactive compounds on cardiovascular organs. Front. Pharmacol..

[68] Yang Y., Xu Q., Li T., Shao S. (2021). Trimetazidine ameliorates hindlimb ischaemic damage in type 2 diabetic mice. Ann. Med..

[69] Wang L., Jiao X.F., Wu C., Li X.Q., Sun H.X., Shen X.Y., Zhang K.Z., Zhao C., Liu L., Wang M. (2021). Trimetazidine attenuates dexamethasone-induced muscle atrophy via inhibiting NLRP3/GSDMD pathway-mediated pyroptosis. Cell. Death Discov..

[70] Farzaei M.H., Ramezani-Aliakbari F., Ramezani-Aliakbari M., Zarei M., Komaki A., Shahidi S., Sarihi A., Salehi I. (2023). Regulatory effects of trimetazidine in cardiac ischemia/reperfusion injury. Naunyn Schmiedebergs Arch. Pharmacol..

[71] Khatana C., Saini N.K., Chakrabarti S., Saini V., Sharma A., Saini R.V., Saini A.K. (2020). Mechanistic insights into the oxidized low-density lipoprotein-induced atherosclerosis. Oxid. Med. Cell. Longev..

[72] Kamisah Y., Che Hassan H.H. (2023). Therapeutic use and molecular aspects of ivabradine in cardiac remodeling: A review. Int. J. Mol. Sci..

[73] Camaré C., Pucelle M., Nègre-Salvayre A., Salvayre R. (2017). Angiogenesis in the atherosclerotic plaque. Redox Biol..

[74] Ouarné M., Pena A., Franco C.A. (2021). From remodeling to quiescence: The transformation of the vascular network. Cells Dev..

[75] Li H., Ma Z., Zhai Y., Lv C., Yuan P., Zhu F., Wei L., Li Q., Qi X. (2020). Trimetazidine ameliorates myocardial metabolic remodeling in isoproterenol-induced rats through regulating ketone body metabolism via activating AMPK and PPARα. Front. Pharmacol..

[76] Fan Y., Yang Q., Yang Y., Gao Z., Ma Y., Zhang L., Liang W., Ding G. (2019). Sirt6 suppresses high glucose-induced mitochondrial dysfunction and apoptosis in podocytes through AMPK activation. Int. J. Biol. Sci..

[77] Chen W., Wu P., Yu F., Luo G., Qing L., Tang J. (2022). HIF-1alpha regulates bone homeostasis and angiogenesis, participating in the occurrence of bone metabolic diseases. Cells.

[78] Shu H.Y., Peng Y.Z., Hang W.J., Zhang M., Shen L., Wang D.W., Zhou N. (2022). Trimetazidine enhances myocardial angiogenesis in pressure overload-induced cardiac hypertrophy mice through directly activating Akt and promoting the binding of HSF1 to VEGF-A promoter. Acta Pharmacol. Sin..

[79] Yang Q., Li S., Zhou Z., Yang X., Liu Y., Hao K., Fu M. (2022). Trimetazidine mitigates high glucose-induced retinal endothelial dysfunction by inhibiting PI3K/Akt/mTOR pathway-mediated autophagy. Bioengineered.

[80] Kim S.G., Sung J.Y., Kim J.R., Choi H.C. (2020). Quercetin-induced apoptosis ameliorates vascular smooth muscle cell senescence through AMP-activated protein kinase signaling pathway. Korean J. Physiol. Pharmacol..

[81] Xiao X.L., Hu N., Zhang X.Z., Jiang M., Chen C., Ma R., Ma Z.G., Gao J.L., Xuan X.C., Sun Z.J. (2018). Niclosamide inhibits vascular smooth muscle cell proliferation and migration and attenuates neointimal hyperplasia in injured rat carotid arteries. Br. J. Pharmacol..

[82] Cao M., Lai P., Liu X., Liu F., Qin Y., Tu P., Wang Y. (2023). ATF5 promotes malignant T cell survival through the PI3K/AKT/mTOR pathway in cutaneous T cell lymphoma. Front. Immunol..

[83] Wang Y., Su H., Yan M., Zhang L., Tang J., Li Q., Gu X., Gong Q. (2020). Interleukin-33 promotes cell survival via p38 MAPK-mediated interleukin-6 gene expression and release in pediatric AML. Front. Immunol..

[84] Grootaert M.O.J., Moulis M., Roth L., Martinet W., Vindis C., Bennett M.R., De Meyer G.R.Y. (2018). Vascular smooth muscle cell death, autophagy and senescence in atherosclerosis. Cardiovasc. Res..

[85] Tian X., Zhou N., Yuan J., Lu L., Zhang Q., Wei M., Zou Y., Yuan L. (2020). Heat shock transcription factor 1 regulates exercise-induced myocardial angiogenesis after pressure overload via HIF-1alpha/VEGF pathway. J. Cell. Mol. Med..

[86] D’Arcy M.S. (2019). Cell death: A review of the major forms of apoptosis, necrosis and autophagy. Cell Biol. Int..

[87] Zhao X., Su L., He X., Zhao B., Miao J. (2020). Long noncoding RNA CA7-4 promotes autophagy and apoptosis via sponging MIR877-3P and MIR5680 in high glucose-induced vascular endothelial cells. Autophagy.

[88] Zhang B., Wan S., Liu H., Qiu Q., Chen H., Chen Z., Wang L., Liu X. (2022). Naringenin alleviates renal ischemia reperfusion injury by suppressing ER stress-induced pyroptosis and apoptosis through activating Nrf2/HO-1 signaling pathway. Oxid. Med. Cell. Longev..

[89] Zhang L., Ding W.Y., Wang Z.H., Tang M.X., Wang F., Li Y., Zhong M., Zhang Y., Zhang W. (2016). Early administration of trimetazidine attenuates diabetic cardiomyopathy in rats by alleviating fibrosis, reducing apoptosis and enhancing autophagy. J. Transl. Med..

[90] Amini N., Sarkaki A., Dianat M., Mard S.A., Ahangarpour A., Badavi M. (2019). The renoprotective effects of naringin and trimetazidine on renal ischemia/reperfusion injury in rats through inhibition of apoptosis and downregulation of micoRNA-10a. Biomed. Pharmacother..

[91] Mustafa N.H., Jalil J., Zainalabidin S., Saleh M.S.M., Asmadi A.Y., Kamisah Y. (2022). Molecular mechanisms of sacubitril/valsartan in cardiac remodeling. Front. Pharmacol..

[92] Dézsi C.A. (2016). Trimetazidine in practice: Review of the clinical and experimental evidence. Am. J. Ther..

[93] Jackson P.J., Brownsill R.D., Taylor A.R., Resplandy G., Walther B., Schwietert H.R. (1996). Identification of trimetazidine metabolites in human urine and plasma. Xenobiotica.

[94] Nenchev N., Skopek J., Arora D., Samad A., Kaplan S., Domahidy M., de Voogd H., Böhmert S., Ramos R.S., Jain S. (2020). Effect of age and renal impairment on the pharmacokinetics and safety of trimetazidine: An open-label multiple-dose study. Drug Dev. Res..

[95] Borowicz-Reutt K., Banach M. (2022). Trimetazidine, an anti-ischemic drug, reduces the antielectroshock effects of certain first-generation antiepileptic drugs. Int. J. Mol. Sci..

